# The efficacy of intravenous aminocaproic acid in primary total hip and knee arthroplasty: a meta-analysis

**DOI:** 10.1186/s13018-018-0802-5

**Published:** 2018-04-17

**Authors:** Yong-jiang Li, Bi-sheng Xu, Sun-peng Bai, Xiao-jun Guo, Xiang-yuan Yan

**Affiliations:** Department of Orthopedics, Tianmen City No.1 People’s Hospital, Tianmen, 431700 Hubei Province People’s Republic of China

**Keywords:** Aminocaproic acid, Arthroplasty, Blood loss, Transfusion, Meta-analysis

## Abstract

**Background:**

We conducted a meta-analysis from randomized controlled trials (RCTs) and non-RCTs to assess the efficacy of aminocaproic acid in cases of primary total hip arthroplasty (THA) or total knee arthroplasty (TKA).

**Methods:**

Potentially relevant academic articles were identified from the Cochrane Library, MEDLINE (1966–2017 October 31), PubMed (1966–2017 October 31), EMBASE (1980–2017 October 31), and ScienceDirect (1985–2017 October 31). Secondary sources were identified from the references of the included literature. The pooled data were analyzed using RevMan 5.1.

**Results:**

Three RCTs and four non-RCTs met the inclusion criteria. There were significant differences in total blood loss (mean difference (MD) = − 495.80, 95% CI − 837.29 to − 154.32, *P* = 0.004), drainage volume (MD = − 249.43, 95% CI − 286.78 to − 212.08, *P* < 0.00001), postoperative hemoglobin level (MD = 0.90, 95% CI 0.78 to 1.02, *P* < 0.00001), hemoglobin reduction (MD = − 0.75, 95% CI − 0.93 to − 0.57, *P* < 0.00001), transfusion rates (risk difference (RD) = − 0.17, 95% CI − 0.25 to − 0.09, *P* < 0.0001), average transfusion units (MD = − 0.28, 95% CI − 0.48 to − 0.09, *P* = 0.004), and length of hospital stay (MD = − 0.33, 95% CI − 0.43 to − 0.24, *P* < 0.00001) between the two groups. No significant differences were found regarding deep vein thrombosis (DVT) (RD = − 0.00, 95% CI − 0.01 to 0.00, *P* = 0.36) between the two groups.

**Conclusions:**

The present meta-analysis indicated that the application of aminocaproic acid in THA or TKA decreases the total blood loss, drainage volume, transfusion rate, transfusion units per patient, and length of hospital stay and does not increase the risk of DVT.

## Background

Total hip arthroplasty (THA) and total knee arthroplasty (TKA) are common treatment for treating the patients suffering end-stage joint disease [1, 2]. It is known that significant blood loss may lead to acute anemia following a THA, and blood transfusions are often required for patients suffering from acute anemia and carry their own risks, such as inducing infectious disease, hemolysis, and anaphylactic reactions and increasing the economic burden [3, 4]. Multiple strategies have been utilized to minimize the blood loss, such as blood salvage, normovolemic hemodilution, electrocautery, hypotensive anesthesia, and hemostatic agents [5]. However, many patients still require blood transfusions.

Various studies have reported that aminocaproic acid reduces blood loss and allogenic blood transfusions in primary THA and TKA [6–12]. However, the results are not consistent. Moreover, some limitations exist in previous studies such as small sample size, inconclusive results, and inaccurate evaluations. Therefore, we conducted a large sample meta-analysis to evaluate the efficacy of aminocaproic acid in primary THA and TKA from randomized controlled trials (RCTs) and non-RCTs.

## Methods

### Search strategy

**Fig. 1:**
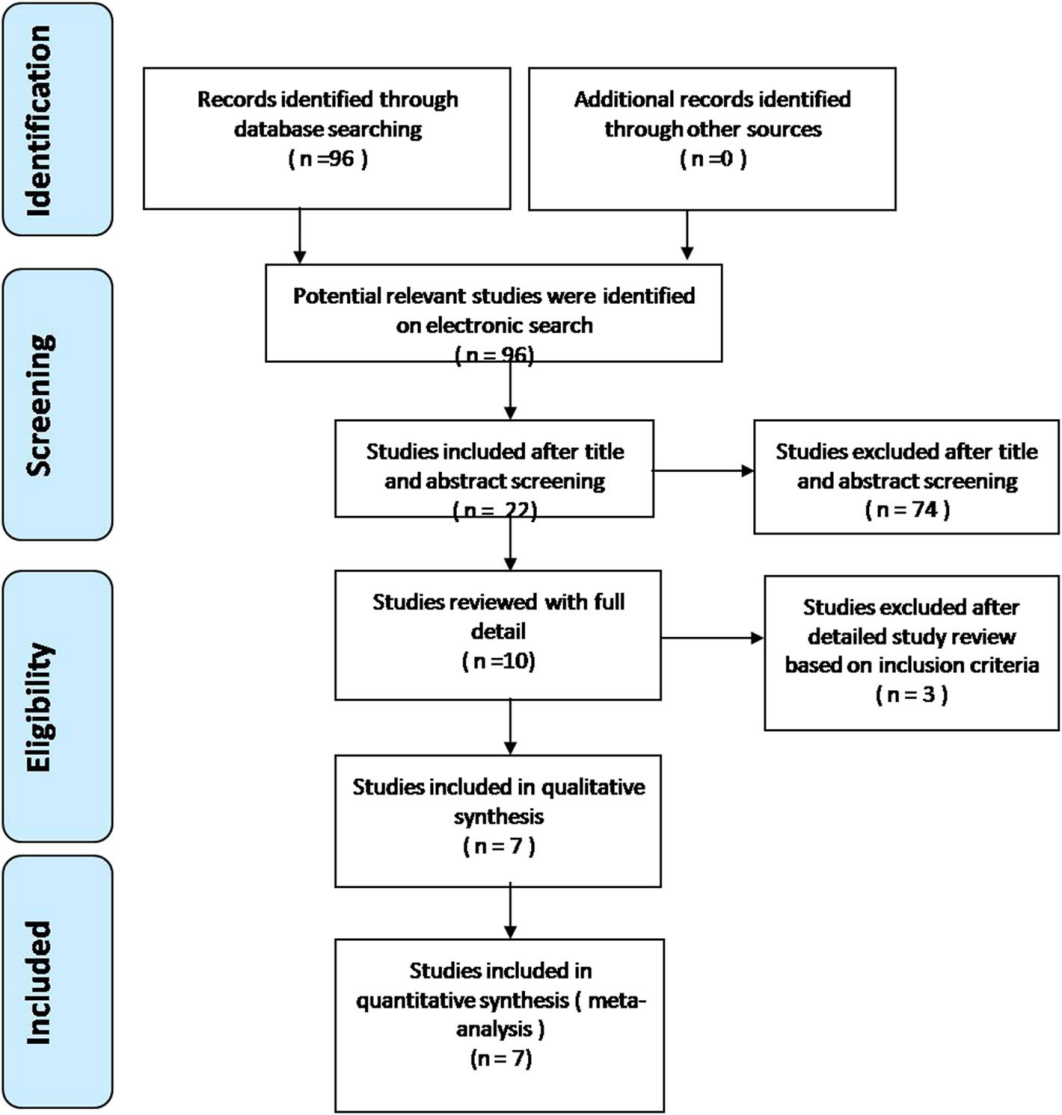
Flow chart of the study selection and inclusion process

Electronic databases were searched, including Cochrane Library, MEDLINE (1966–2017 October 31), PubMed (1966–2017 October 31), EMBASE (1980–2017 October 31), and ScienceDirect (1985–2017 October 31). We then manually searched the reference lists of all included studies, relevant books, review articles, and meeting proceedings to identify trials that might have been missed in the electronic search. The search process was conducted as follows in Fig. 1. The key words “aminocaproic acid” and “replacement OR arthroplasty” were used in combination with the Boolean operators AND or. We made no restrictions on the language of the publication. This study is a meta-analysis and did not need approval from the ethics committee or institutional review board.

### Inclusion criteria

Studies were considered eligible for inclusion if they met the following criteria: (1) patients undergoing a primary THA or TKA; (2) the intervention used aminocaproic acid and studies contained a control group with placebo or null; (3) the outcomes included blood loss, operative time, blood transfusion rate, blood transfusion unit, perioperative outcomes, and complications; and (4) the study was a published or unpublished comparative trial (RCTs or non-RCTs).

### Exclusive criteria

We excluded articles that were (1) studies without controlled groups, (2) articles without available full-text versions, and (3) no available outcomes data.

### Selection criteria

Two reviewers independently screened the titles and abstracts for eligibility criteria. Subsequently, the full text of the studies that potentially met the inclusion criteria were read, and the literature was reviewed to determine final inclusion. Disagreement was resolved by consulting a third reviewer.

### Quality assessment

According to whether the study is a randomized or non-randomized trial, the methodological Index for Non-randomized Studies (MINORS) form was used to assess retrospective controlled trials [13]. Quality assessment for RCT was conducted according to a modification of the generic evaluation tool used by the Cochrane Bone, Joint and Muscle Trauma Group [14]. Disagreements were resolved by consensus or consultation with the senior reviewer.

### Data extraction

Two researchers independently extracted the data from the included literature. The corresponding author was consulted for details in the case of incomplete data. The following information was extracted: first author name, year of publication, intervening measures, comparable baseline, sample size, and outcome measures. We contacted the authors of the studies for further information. Other relevant parameters were also extracted from individual studies.

### Data analysis and statistical methods

Pooling of data was analyzed by RevMan 5.1 (The Cochrane Collaboration, Oxford, UK). Heterogeneity was estimated depending on the value of *P* and *I*^2^ using the standard chi-square test. When *I*^2^ > 50%, *P* < 0.1 was considered to be significant heterogeneity. Therefore, a random effects model was applied for data analysis. A fixed effects model was used when no significant heterogeneity was found. Subgroup analysis was performed to investigate sources in the case of significant heterogeneity. Mean difference (MD) and 95% confidence interval (CI) were presented for continuous outcomes. Risk difference (RD) and 95% CIs were calculated for dichotomous data.

## Results

### Search results

A total of 96 studies were identified as potentially relevant literature reports. No additional studies were obtained after the reference review. Seventy-four studies were excluded after title and abstract screening, 22 studies reviewed with full detail, and 15 of studies excluded after detailed study review based on inclusion criteria. Ultimately, three RCTs and four non-RCTs were eligible for data extraction and meta-analysis. The search process is shown in Fig. 1.

### Risk of bias assessment

**Fig. 2:**
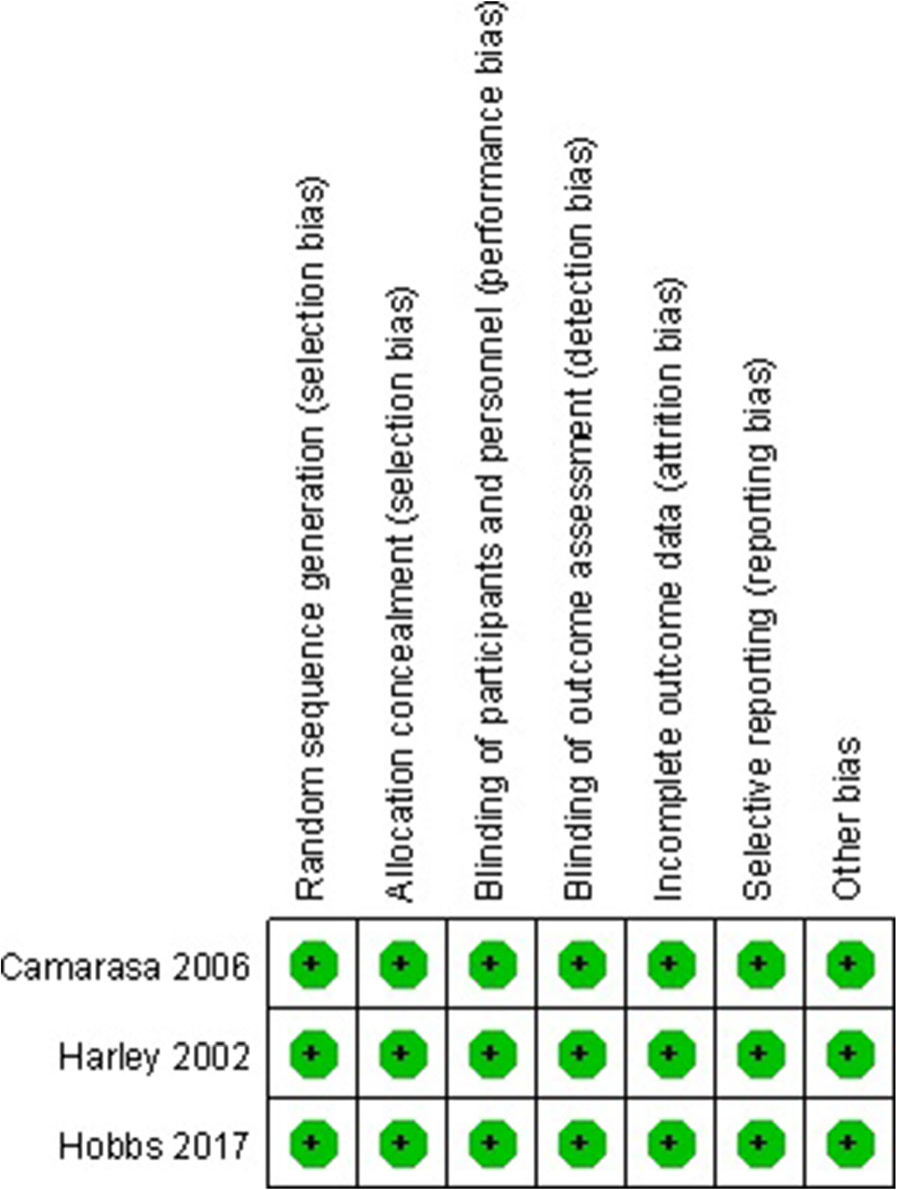
The summary of bias risk of randomized controlled trials

RCT quality was assessed based on the Cochrane Handbook for Systematic Review of Interventions (Fig. 2). The RCT stated clear inclusion and exclusion criteria. Included RCT performed adequate methodology of randomization, concealment of allocation, blinding, and intent-to-treatment analysis. No unclear bias was reported due to incomplete outcome data or selective outcomes. For the non-RCTs, the MINORS scores were 18–20 for the retrospectively controlled trials. The methodological quality assessment is illustrated in Table 1.

### Study characteristics

Demographic characteristics and details concerning the literature type of the included studies are summarized in Table 2. Statistically similar baseline characteristics were observed between both groups.

**Table 2:** Characteristics of included studies

Study	Operation	Cases (A/C)	Mean age (A/C)	Gender (F)	Dosage	Prophylactic anticoagulant	Transfusion trigger
Camarasa et al. 2006	TKA	32/60	73/72	28/48	100 mg/kg administered intravenously in 30 min (before tourniquet release) + 3 g for 3 h following first dose	LMWH	Hb < 8 g/dl or 10 g/dl with clinical symptoms
Churchill et al. 2017	TKA	820/1492	63.9/63.9	527/956	5 g (BW < 50 kg); 10 g (BW > 50 kg) administered intravenously near the time of tourniquet release	Surgeon’s discretion	NS
Churchill et al. 2016 (THA)	THA	911/643	65.1/65.4	392/377	5 g (BW < 50 kg); 10 g (BW > 50 kg) administered intravenously near the time of incision	Surgeon’s discretion	Surgeon’s discretion
Churchill et al. 2016 (TKA)	TKA	25/25	65.2/66.6	21/15	10 g administered intravenously over 10 min and was completely infused before tourniquet deflation	Warfarin	Hb < 7 g/dl or 9 g/dl with clinical symptoms
Harley et al. 2002	THA	26/29	69/69	16/18	150 mg/kg administered intravenously over 20 min on the patient’s arrival in the operating room + 12.5 mg/kg/h for an additional 5 h	Heparin	Hb < 80 g/L or HCT < 0.24 or patients having anemia symptoms
Hobbs et al. 2017	THA and TKA	184/185	62.1/63.1	14/14	5 g administered intravenously over 20 min before incision + 5 g again during closure	Asprin, LMWH	Surgeon’s discretion
Ray et al. 2005	THA	15/15	72/69	NS	10 g administered intravenously over 30 min after the induction of anesthesia + 5 g over 3 h	Aspirin	NS

*THA* total hip arthroplasty, *TKA* total knee arthroplasty, *A* aminocaproic acid, *C* control, *F* female, *BW* body weight, *NS* not state, *M* male, *LMWH* low molecular weight heparin, *HCT* hematocrit, *Hb* hemoglobin

**Table 1:** Quality assessment for non-randomized trials

Quality assessment for non-randomized trials	Churchill et al. 2016 (THA)	Churchill et al. 2016 (TKA)	Churchill et al. 2017	Hobbs et al. 2017
A clearly stated aim	2	2	2	2
Inclusion of consecutive patients	2	2	2	2
Prospective data collection	0	0	0	0
Endpoints appropriate to the aim of the study	2	2	2	2
Unbiased assessment of the study endpoint	2	2	2	2
A follow-up period appropriate to the aims of study	2	2	2	2
Less than 5% loss to follow-up	2	2	2	2
Prospective calculation of the sample size	0	0	0	0
An adequate control group	2	2	2	2
Contemporary groups	2	2	2	0
Baseline equivalence of groups	2	2	2	2
Adequate statistical analyses	2	2	2	2
Total score	20	20	20	18

**Table 3:** Subgroup analysis of blood transfusion rate

Outcome of subgroup	Studies	Effect estimate				
		*χ* ^2^	*I*^2^ (%)	RD	95% CI	*P* value
TKA	3	13.34	88	− 0.22	[− 0.42, − 0.02]	0.03
THA	3	3.20	38	− 0.17	[− 0.21, − 0.13]	0.00001
Transfusion trigger	3	4.47	57	− 0.23	[− 0.35, − 0.12]	0.0001
Continuous application	4	4.88	39	− 0.20	[− 0.26, − 0.14]	0.00001

*TKA* total knee arthroplasty, *THA* total hip arthroplasty, *CI* confidence interval, *MD* mean difference

### Outcomes of meta-analysis

#### Total blood loss

**Fig. 3:**
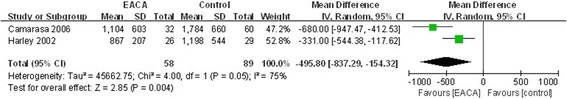
Forest plot of total blood loss

Two of the included articles reported the outcomes for total blood loss [10, 12]. There was significant heterogeneity (*χ*^2^ = 4.00, df = 1, *I*^2^ = 75%, *P* = 0.05); as a result, a random model was applied. The pooled results demonstrated the total blood loss in the aminocaproic acid group was significantly lower than that in the control group (MD = − 495.80, 95% CI − 837.29 to − 154.32, *P* = 0.004; Fig. 3).

#### Drainage volume

**Fig. 4:**
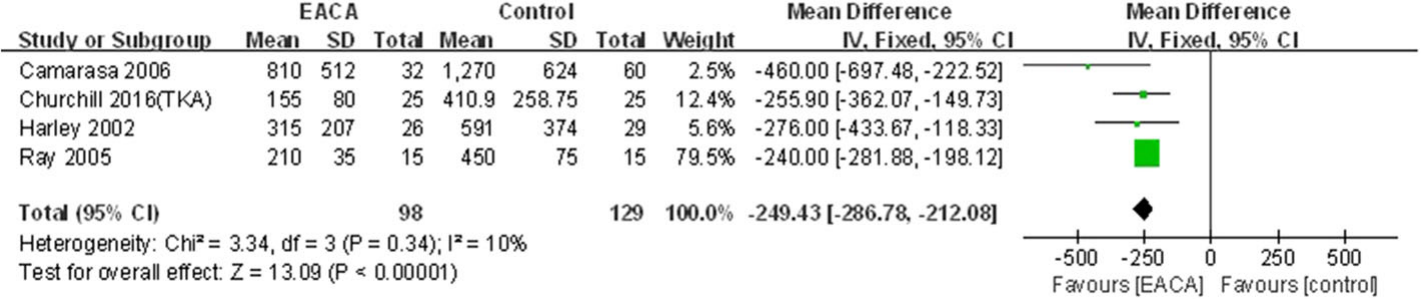
Forest plot of drainage volume

Drainage volume was reported in four included studies [8, 10–12]. No significant heterogeneity was found, a fixed model was applied (*χ*^2^ = 3.34, df = 3, *I*^2^ = 10%, *P* = 0.34). The differences between the two groups was statistically significant (MD = − 249.43, 95% CI − 286.78 to − 212.08, *P* < 0.00001; Fig. 4).

#### Postoperative hemoglobin level

**Fig. 5:**
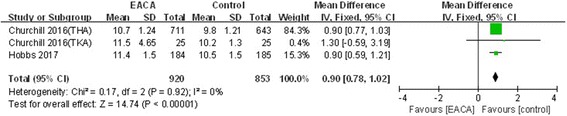
Forest plot of postoperative hemoglobin level

Three included studies reported postoperative hemoglobin level [6, 8, 9]. There was no significant heterogeneity (*χ*^2^ = 0.17, df = 2, *I*^2^ = 0%, *P* = 0.92); as a result, a fixed model was applied. The pooled results demonstrated the postoperative hemoglobin level in the aminocaproic acid group was significantly higher than in the control group (MD = 0.90, 95% CI 0.78 to 1.02, *P* < 0.00001; Fig. 5).

#### Hemoglobin reduction

**Fig. 6:**
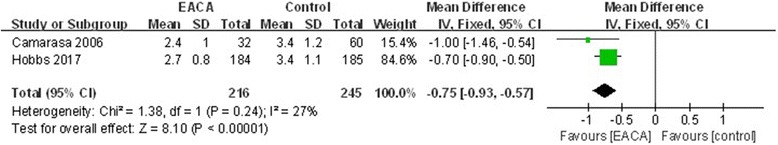
Forest plot of hemoglobin reduction

Hemoglobin reduction was reported in two included studies [6, 10]. No significant heterogeneity was found, a fixed model was applied (*χ*^2^ = 1.38, df = 1, *I*^2^ = 27%, *P* = 0.24). The differences between the two groups was statistically significant (MD = − 0.75, 95% CI − 0.93 to − 0.57, *P* < 0.00001; Fig. 6).

#### Blood transfusion rate

**Fig. 7:**
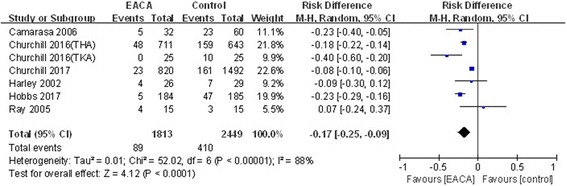
Forest plot of blood transfusion rate

The blood transfusion rate was reported in seven included studies [6–12]. A random model was employed, which significant heterogeneity was found (*χ*^2^ = 52.02, df = 6, *I*^2^ = 88%, *P* < 0.00001). The difference between the two groups in regard to the blood transfusion rate was statistically significant (RD = − 0.17, 95% CI − 0.25 to − 0.09, *P* < 0.0001; Fig. 7).

A subgroup analysis was performed for the blood transfusion rate, showing that this positive effect persisted regardless of the delivered dosage, whether the patient had received TKA or THA, and whether a transfusion protocol existed (Table 3).

#### Transfusion units per patient

**Fig. 8:**
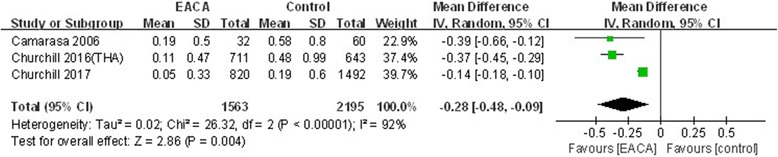
Forest plot of transfusion units per patient

Three included studies reported the outcome of the transfusion units per patient [7, 9, 10]. The random model was employed according to a significant heterogeneity (*χ*^2^ = 26.32, df = 2, *I*^2^ = 92%, *P* < 0.00001). There were statistically significant differences between the two groups (MD = − 0.28, 95% CI − 0.48 to − 0.09, *P* = 0.004; Fig. 8).

#### Deep vein thrombosis (DVT)

**Fig. 9:**
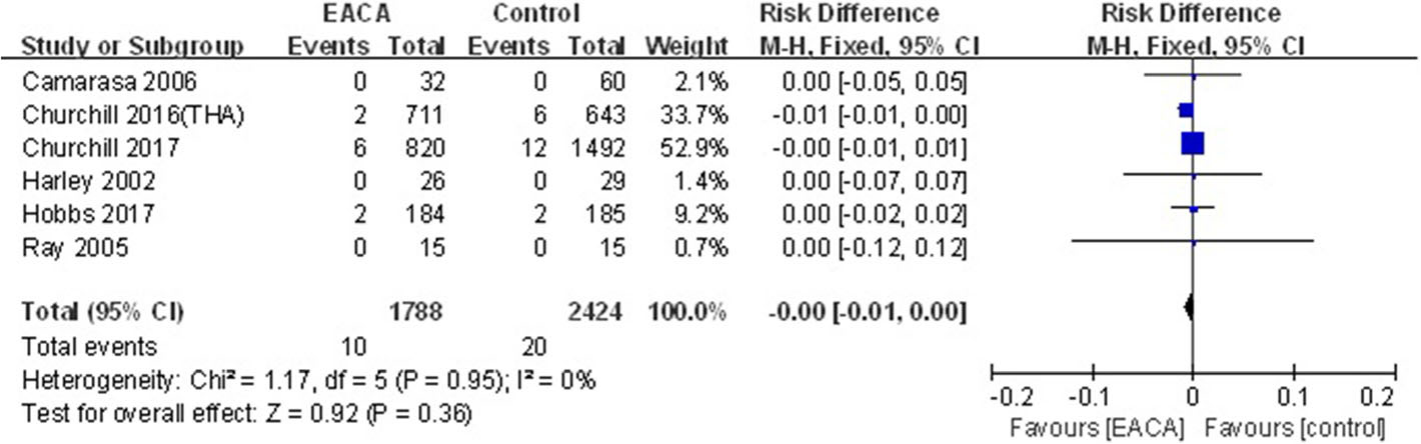
Forest plot of deep vein thrombosis

The incidence of DVT had been reported in six studies [6, 7, 9–12]. The low significant heterogeneity was found, a fixed model was applied (*χ*^2^ = 1.17, df = 5, *I*^2^ = 0%, *P* = 0.95). No significant differences between the groups were found (RD = − 0.00, 95% CI − 0.01 to 0.00, *P* = 0.36; Fig. 9).

#### Length of hospital stay

**Fig. 10:**
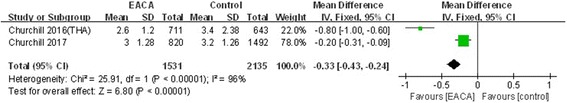
Forest plot of length of hospital stay

Two studies reported the length of hospital stay [7, 9]. There was significant heterogeneity shown between the pooled results; therefore, a random model was applied (*χ*^2^ = 25.91, df = 1, *I*^2^ = 96%, *P* < 0.00001). There was a significant difference of length of hospital stay between the groups (MD = − 0.33, 95% CI − 0.43 to − 0.24, *P* < 0.00001; Fig. 10).

## Discussion

The most important results of the present meta-analysis were that the application of aminocaproic acid during a THA and TKA decreased total blood loss, drainage volume, transfusion rate, transfusion units per patient, and length of hospital stay and does not increase the risk of DVT. Moreover, the length of hospital stay was shortened when aminocaproic acid was administered intravenously.

Aminocaproic acid, an antifibrinolytic drug, competitively block the lysine-binding site of plasminogen and has been used to reduce blood loss in surgery for many years [15]. The effectiveness of aminocaproic acid for decreasing perioperative blood loss during THA and TKA is widely reported. Present meta-analysis indicated that the intravenous application of aminocaproic acid could significantly decrease total blood loss and drainage volume. These results are similar to those of RCTs [10–12].

The indications for blood transfusion were based on hemoglobin levels and clinical symptoms of anemia [16]. Several studies have demonstrated that aminocaproic acid could reduce postoperative Hb reduction [6, 10]. Our meta-analysis was consistent with these results. Pooled result also showed that postoperative hemoglobin level in the aminocaproic acid group was significantly higher than that in the control group. Although transfusion trigger varied from included studies, present meta-analysis indicates that the application of aminocaproic acid significantly decrease the blood transfusion rate and the average transfusion units. An RCT reported by Ray et al. [11] showed that aminocaproic acid does not reduce transfusion requirements. Their studies did not report the transfusion trigger.

Theoretically, antifibrinolytic agents may increase the risk of thrombotic events [17]. DVT is a common complication in orthopedic surgery, especially in arthroplasty, and may progress to pulmonary embolism and even death [18, 19]. All included studies reported the use of an anticoagulant therapy after surgery. The meta-analysis showed that the use of aminocaproic acid did not increase the risk of DVT, which was 0.55% with the aminocaproic acid and 0.83% in the controls.

Several potential limitations should be noted. (1) Only seven studies were included, all of which had a relatively small sample size; (2) methodological weaknesses exist in all included studies, and some outcome parameters were not fully described so that we failed to perform a meta-analysis; and (3) subgroup analysis was not performed because of the limited number of included studies, and we could not determine the source of heterogeneity.

## Conclusions

The present meta-analysis indicated that the application of aminocaproic acid in THA and TKA decreases the total blood loss, drainage volume, transfusion rate, transfusion units per patient, and length of hospital stay and does not increase the risk of DVT or other complications.

## Abbreviations

CI: Confidence interval
DVT: Deep vein thrombosis
MD: Mean difference
MINORS: Methodological Index for Non-randomized Studies
RCTs: Randomized controlled trials
RD: Risk difference
THA: Total hip arthroplasty
TKA: Total knee arthroplasty
